# The value of banknotes: relevance of size, colour and design

**DOI:** 10.1007/s00426-022-01764-x

**Published:** 2022-11-15

**Authors:** Fernando Ojedo, Pedro Macizo

**Affiliations:** 1 Departamento de Psicología Experimental, Facultad de Psicología, Universidad de Granada, Campus de Cartuja, s/n., 18071 Granada, Spain; 2 Mind, Brain and Behaviour Research Center (CIMCYC), Granada, Spain

## Abstract

In the current study, we evaluate the relevance of three physical features when people retrieve the monetary value of banknotes. To this end, three monetary comparison tasks were designed in which in each trial a pair of banknotes were presented and participants selected the one with higher monetary value. In each task, a different banknote feature (size, colour and design) was examined and a congruent and an incongruent condition (the value of the physical feature corresponded or not to its actual value, respectively) were compared to a neutral condition (no information about the physical feature was provided). We found a pattern of facilitation and interference effects which suggests that size is the most relevant physical feature for accessing the monetary value of banknotes followed by colour. However, the availability of a variety of designs across banknotes seemed not to facilitate the performance of the task, but rather the opposite, hindering the monetary comparison task.

## Introduction

Dealing with money is a routine activity involved in a multitude of tasks that people perform in everyday life. For example, in 2016, consumers averaged 1.2 cash transactions per day (Esselink & Hernandez, 2017). These monetary tasks are usually carried out with different formats of money such as prices, coins and bills. At first, people could handle money properly attending to only the monetary category and the number imprinted on the cash. For example, 10 dollars is more money than 10 cents if we focus on the monetary category (i.e., the economic value of the dollar > cent), and 10 dollars is more money than 1 dollar if we consider the numerical magnitude (10 > 1). However, the handling of cash is not an easy activity. People have different biases when dealing with money (e.g., Coulter & Coulter, 2005). For instance, the processing of physical features of currency such as format (e.g., coins vs. bills) and size leads to errors when people evaluate sums of money (Goldman et al., 2012; Hasegawa, 2020; Peetz & Soliman, 2016).

To illustrate, with regard to *price* processing, individuals perceive greater discounts on price pairs (current price/reduced price) containing smaller units (e.g., $23/$22) relative to prices with larger units (e.g., $19/$18) even though the discount is the same in both cases (i.e., $1) (Coulter & Coulter, 2005). In addition, when people compare price pairs (e.g., 2 euros > 9 cents), the processing of the numbers may be misleading when the higher price contains a smaller number than the lower price (2 euros > 9 cents but 2 < 9) relative to price pairs in which the monetary category and the number lead to the same decision (9 euros—2 cents, where euros > cents and 9 > 2) (Cao et al., 2015; Macizo & Ojedo, 2018; Ojedo & Macizo, 2020). Thus, people have processing biases such as the “illusion of money” under which one hundred cents appears greater than one dollar (Shafir et al., 1997). In addition, the physical format of prices also influences the evaluation of their monetary value. For example, people are less efficient at comparing prices when their physical size is incongruent with their economic value (e.g., $12–$10) versus a congruent situation in which their physical size is in line with their monetary value (e.g., $12–$10) (Coulter & Coulter, 2005).

On the other hand, *coins* are representations of amounts of money that differ in physical features such as size and colour. It has been observed that, in general, coins are designed to favour the distinctiveness between them and the monetary value they reflect (e.g., Pavlek et al., 2020). Regarding the physical features of coins, several studies have evaluated the relationship between size and monetary value (Fitousi, 2010; Goldman et al., 2012; Hasegawa, 2020; Peetz & Soliman, 2016). Goldman et al. showed that when people have to judge the monetary value of coins in the Israeli currency (the shekel, sh), the performance is less efficient when the size of the coins is inconsistent with their economic value (5–10sh) compared to situations where one coin is larger than another in both size and value (1–5sh). Thus, the physical size of the coins affects the evaluation of the amount of money they represent. Moreover, Peetz and Soliman (2016) showed that the physical size of coins makes people overvalue them. In their study, the authors used coins in the Canadian currency that were enlarged by 15% of their actual size. Individuals rated these oversized coins as more valuable than coins displayed in the real size. Thus, people seem to overestimate the value of coins where greater size is interpreted as greater value (i.e., the “bigger is better” heuristic, Silvera et al., 2002). In turn, Hasegawa (2020) revealed the inverse relationship, that is, how the monetary value of coins modulates the estimation of their size (e.g., more vs. less valuable coins are judged as larger on size, Leiser & Izak, 1987; see also, den Daas et al., 2013; Dubois et al., 2010). In his study, Hasegawa selected two coins in the Japanese currency with equal physical size but different monetary value (10–100 yen). The size of these coins was edited to implement a congruent size condition in which the 100 yen coin was larger than the 10 yen coin and an incongruent condition where the 100 yen coin was smaller than the 10 yen coin. Individuals displayed worse performance in the incongruent condition than in the congruent condition indicating that they retrieved the monetary value while estimating the size of the coins.

On the other hand, there is recent research on how people perceive and produce the monetary value of *banknotes* (Di Muro & Noseworthy, 2013; Giuliani et al., 2018; Macizo & Herrera, 2013; Macizo & Morales, 2015; Manippa et al., 2019; Mishra et al., 2006; Raghubir & Srivastava, 2009; Ruiz et al., 2017). Banknotes represent amounts of money that people can identify with accuracy based on the monetary category and the number depicted on each bill. However, different factors modulate the amount of money that people attribute to banknotes in their daily lives. For example, people judge with less economic value the bills that seem used and shabby compared to bills that, despite representing the same amount of money, appear crisp and new (Di Muro & Noseworthy, 2013). In addition, people are more likely to spend money when the same economic amount is presented in smaller bills than when it is shown in a single bill of greater size (Mishra et al., 2006; Raghubir & Srivastava, 2009). Moreover, people tend to attribute greater economic value to bills that are more familiar to them (e.g., regular $1 bill) compared to less familiar bills (e.g., rare $2 bill) (Alter & Oppenheimer, 2008). On the other hand, the recognition of banknotes seems to depend on the visual field in which they are displayed. Giuliani et al. (2018) showed that 100 banknotes are recognized faster than 5 banknotes when these bills are presented in the right visual field while no differences are observed between the processing of bills when they are presented in the left visual field. The results were interpreted on the basis of the association between the positive valence of higher value banknotes and the positive valence of the right visuospatial side, indicating also the possible specific peculiarities that the money may have when it is processed. Finally, the physical size determines the processing of banknotes in monetary comparison tasks as occurred with the processing of prices (Coulter & Coulter, 2005) and coins (Goldman et al., 2012). Thus, in a monetary comparison task with pairs of banknotes, the selection of the bill with higher economic value depends on the congruency between its value and physical size (Ruiz et al., 2017). Thus, Ruiz et al. showed that monetary comparisons are more efficient when there is congruency between the value and size of euro banknotes (e.g., 20–100€ banknote pair in which the ratio of the bills’ physical sizes was preserved) compared to pairs of banknotes in which their physical size was equated (e.g., 20–100€ banknote pair presented with the same size).

Therefore, physical features such as size determine how people estimate the economic value of banknotes (e.g., Ruiz et al., 2017). However, in many currencies (euro currency, US dollar, Canadian dollar, British pound, Indian rupee, Chinese renminbi, etc.), the bills differ in other features such as colour and design (i.e., image printed on them). The objective of this study was to evaluate the relevance of the physical features of banknotes (size, colour and design) when individuals determine their monetary value. To this end, we used a monetary comparison task in which pairs of banknotes were presented and individuals selected the one with higher monetary value. The task was done with euro banknotes (5€, 10€, 20€, 50€, 100€). These banknotes differ proportionally in size (120 × 62 mm, 127 × 67 mm, 133 × 72 mm, 140 × 77 mm and 147 × 82 mm, respectively), main colour (grey, red, blue, orange and green, respectively) and design (doors, windows and bridges of classical, Romanesque, Gothic, Renaissance and Baroque architecture, respectively).

**Fig. 1:**
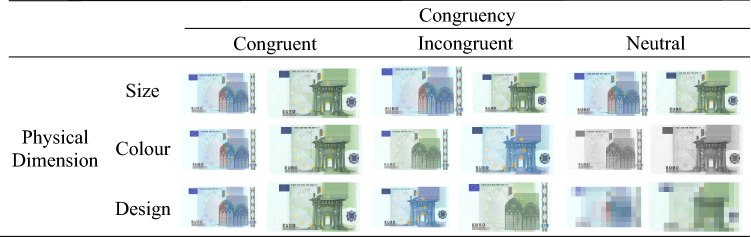
Example of trials in each experimental condition

In our study, we developed three banknote Stroop-like comparison tasks to evaluate the processing of the size, colour and design of banknotes. Three experimental conditions were used in each task (see Fig. 1). In the congruent condition, the value of the three features (size, colour and design) was the same as that of the banknotes in real life. In the neutral condition, one of the physical features was cancelled so that the value of this feature was not informative of the economic value of banknotes (e.g., in the size version of the banknote comparison task the size of the banknote pair was matched; in the colour version both banknotes were presented in the same grey colour; in the design version the images of the pair of banknotes were pixelated). Finally, in the incongruent condition, the value of one of the features was exchanged between the bills of the pair (e.g., in the size version of the banknote comparison task, a 20€ banknote with the size of a 100€ banknote and a 100€ banknote with the size of a 20€ banknote; in the colour version of the banknote comparison task, a 20€ banknote with the colour of a 100€ banknote and a 100€ banknote with the colour of a 20€ banknote; in the design version, a 20€ banknote with the design of a 100€ banknote and a 100€ banknote with the design of a 20€ banknote). Through this manipulation, we expected to be able to answer two different issues, first, whether the physical features of the banknotes were relevant for the access to the value of the banknotes, and second, to be able to compare them and find out which one plays a more relevant role in the access to the monetary value of the banknotes. In case a physical feature (e.g., size) was informative of the monetary value of banknotes, we expect to observe a facilitation effect with better comparison of the banknotes’ value in the congruent condition, in which this feature was informative (e.g., size ratio equal to the current size of euro banknotes), as opposed to the neutral condition in which the feature was not informative (e.g., banknotes of equal size). In addition, in case people would automatically process the physical features of banknotes, we expect to find an interference effect with worse performance in the incongruent condition, in which the value of that feature was incorrect compared to the neutral condition.

Critically, the magnitude of the facilitation and interference effects in the three versions of the banknote comparison task (size, colour and design) would indicate the relative importance of each feature when individuals evaluate the amount of money represented on each banknote. A priori, we expect that physical size would be the most relevant feature, because size as well as monetary value represent magnitude information (physical and economic magnitudes, respectively). On the other hand, we expect colour to be more relevant than design for two reasons: (a) the differences in the main colour between pairs of banknotes are easily noticed. On the contrary, the comparison of the design of banknote pairs requires a careful analysis of the details printed on each banknote (e.g., to perceive architectural differences between the classical gate and the Romanesque gate represented on the 5€ and 10€ banknotes, respectively); (b) the colour of the euro banknotes has been a consistent feature while their design has undergone variations over time. Thus, while the colour of the euro banknotes has remained the same since the currency came into circulation (year 2002), the design of banknotes has shown slight differences in the architectural elements imprinted on them (e.g., Europe series that came into circulation in 2013). However, this should not necessarily mean that the information provided by the content of the banknote design cannot be useful for accessing the value of them, as it would be possible to identify the design associated with each of the different banknotes. In this regard, it should be noted that the present study was carried out with a specific currency and with a population that used it in their daily lives, since as mentioned above, depending on the currency, the banknotes are distinguishable or not by the features explored in this research. Therefore, the familiarity of the participants with the currency used in the study should play a significant role, as well as in Macizo and Morales (2015), where familiarity was observed to be a key factor in the way banknotes are processed. Therefore, it would be expected that in a task such as the present one where the monetary value of the banknotes has to be accessed, the effect produced by the manipulation of the size would be consistent across currencies (both are continuous magnitudes), but the effect of colour and design would be dependent on previous experience with the currencies under consideration.

## Method

### Participants

Sixty participants (51 women and 9 men) with mean age of 21.68 years (SD = 2.95) took part in the experiment. All participants used the Euro currency on a daily basis. The participants signed an informed consent form before conducting the experiment and they received university credits for participating in the study. The sample size was computed using G*Power program 3.1.9.4 (Faul et al., 2007). It was calculated that for a 3 × 3 multivariate analysis of variance (MANOVA) to achieve 80% statistical power with *α* = 0.05 and an effect size of 0.25, the total sample size needed was *N* = 54. Thus, the number of participants who took part in the experiment was enough to capture the possible effects evaluated in our study.

### Task

The stimuli and experimental task used in the study are fully and freely accessible at https://osf.io/53fpv/?view_only=3f893b40a2094a4e96f7e713678dc151

The experiment was designed and controlled by the experimental software E-Prime 2.0 (Schneider et al., 2002). We developed three banknote Stroop-like comparison tasks where, in each trial, a pair of banknotes were presented on the screen and participants had to indicate the banknote with higher monetary value. The only difference between the three tasks was the physical feature that was manipulated (size, colour and design). All the participants completed the three tasks, and the order in which they received these tasks was counterbalanced across participants.

In the study, the 5€, 10€, 20€, 50€ and 100€ banknotes were used. The Arabic numbers denoting the monetary value were removed from each banknote to prevent participants from performing the comparison task based on the numerical information only.

Three experimental conditions were implemented in each task (see Fig. 1). In the congruent condition, the banknotes had the same size, colour and design as the actual euro banknotes. In the incongruent condition, the banknote pairs of each trial exchanged the value of the feature under study (size, colour or design). For example, a 20€ bill in green shades and a 100€ bill in blue shades (i.e., incongruent condition in the colour version of the banknote Stroop task). Finally, in neutral trials, the pairs of bills had equal value in the feature under study (i.e., same dimension in the size task, grey in the colour task, pixelated image in the design task) while maintaining the actual values of banknotes in the other two features.

In the three versions of the banknote comparison task, pairs of euro bills were presented, one on the right and one on the left of the screen. Each euro banknote used in the study (5€, 10€, 20€, 50€ and 100€) was paired with the rest of banknotes, forming ten possible combinations of banknotes (5–10€, 5–20€, 5–50€, 5–100€, 10–20€, 10–50€, 10–100€, 20–50€, 20–100€, 50–100€). These banknote pairs were presented twice to counterbalance the display layout (left and right) and the monetary value of banknotes (higher, lower). Thus, on ten occasions, the banknote with the higher monetary value was displayed on the right (e.g., 5–10€) and on ten other occasions on the left (e.g., 10€–5€). These 20 pairs of banknotes were presented in the congruent, incongruent and neutral condition at random. Thus, each participant received 60 banknote pairs in each comparison task (size, colour and design version of the task).

### Procedure

Participants were tested individually, seated 60–70 cm approximately from the computer screen (Capture E1903D, LCD, 1280 × 1024, 60 Hz, 19″). In each trial, a pair of banknotes was presented in the middle of the screen and participants were instructed to select the one with higher monetary value, as quickly as possible but without making errors, by pressing the Z or M key of the keyboard if the higher value banknote was located on the left or right side of the screen, respectively. The banknotes remained on the screen until the participants’ response. The interval between trials lasted 300 ms (blank screen). Before starting each of the three versions of the banknote comparison task, the participants performed 5 practice trials. The duration of the experiment was approximately 45 min.

## Results

All data and analyses of the present study are fully and freely available at the following link: https://osf.io/53fpv/?view_only=3f893b40a2094a4e96f7e713678dc151

One participant was excluded from the analyses due to the high error rate (> 50% of the trials). Trials in which participants committed an error were excluded from the latency analysis and submitted to the error rate analysis, the percentage of errors was: 5.51% in the size-based task, 3.80% in the colour-based task, and 4.58% in the design-based task. Afterwards, the reaction times (RTs) associated with correct responses were trimmed following the procedure described by Tabachnick and Fidell (2007) to eliminate univariate outliers. Raw scores were converted to standard scores (*z* scores). Data points which, after standardization, were 3 SD outside the normal distribution, were considered outliers. After removing outliers from the distribution, *z* scores were calculated again. The filter was applied in recursive cycles until no observations were outside 3 SD. The percentage of outliers was 5.53% in the size-based task, 7.14% in the colour-based task, and 7.24% in the design-based task.

**Fig. 2:**
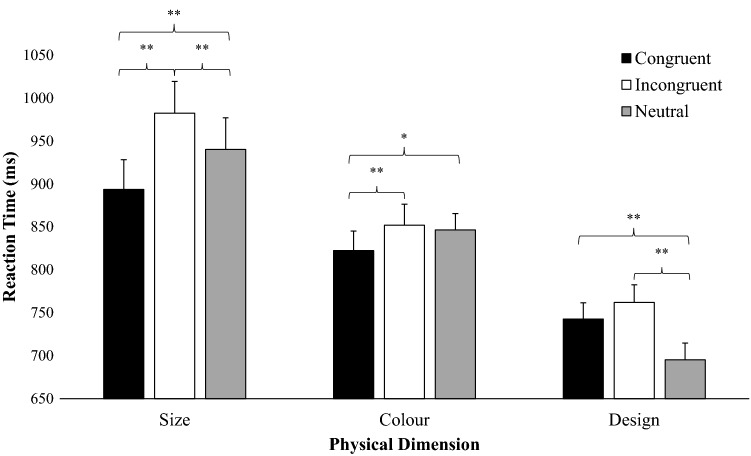
Reaction times (RTs in milliseconds, ms) obtained in each of the three Stroop-like banknote comparison tasks

The RTs and error rates were submitted to an analysis of variance (ANOVA) with congruency (congruent, incongruent and neutral) and banknote feature (size, colour and design) as within-participant factors. The Greenhouse–Geisser correction (Greenhouse & Geisser, 1959) for non-sphericity of variance was used for all *F*-ratios with more than one degree of freedom in the denominator; reported here are the original *df*, the corrected probability level, and the *ε* correction factor. In all analyses reported in text, the critical *p* level for significance was *α* = 0.05. The outcomes of these analyses are reported in Table 1 and Fig. 2. Additional analyses conducted with the order in which the three comparison tasks were performed revealed that this factor did not interact with congruency. Furthermore, the Order x Congruency x Banknote feature three-way interaction was not significant so the order of administration of the tasks was not considered any further.

**Table 1:** Facilitation and interference effect across banknote feature

	Congruent		Incongruent		Neutral		Facilitation	Interference
	RT	E%	RT	E%	RT	E%		
Size	894 (19)	3.81 (0.71)	983 (21)	7.33 (1.69)	940 (19)	4.45 (0.74)	47**	− 42**
Colour	822 (35)	1.73 (0.34)	852 (37)	4.58 (1.00)	846 (37)	1.8 (0.37)	24*	− 6^ns^
Design	743 (23)	2.67 (0.50)	762 (24)	5.68 (1.76)	695 (19)	1.91 (0.35)	− 47**	− 67**

Reaction times (RT) (in milliseconds), error percentage (E%) and standard error (in parentheses) obtained across the physical features of banknotes in the congruent, incongruent and neutral condition. Facilitation = Neutral minus Congruent (RT). Interference = Neutral minus Incongruent (RT)

^ns^*p* > 0.05, *p < 0.01,**p < 0.001

The main effect of congruency was significant in the latency analysis, *F*(2, 116) = 50.24, *p* < 0.001, *ε* = 0.97, *η*^2^ = 0.46, and the error rate analysis, *F*(2, 116) = 12.55, *p* < 0.001, *ε* = 0.57, *η*^2^ = 0.18. The main effect of banknote feature was significant in the latency analysis, *F*(2, 116) = 28.59, *p* < 0.001, *ε* = 0.81, *η*_*p*_^2^ = 0.33, and the error rate analysis, *F*(2, 116) = 4.26, *p* = 0.024, *ε* = 0.80, *η*_*p*_^2^ = 0.07. The Congruency x Banknote feature interaction was significant in the latency analysis, *F*(4, 232) = 19.10, *p* < 0.001, *ε* = 0.84, *η*_*p*_^2^ = 0.25, but not in the error rate analyses, *F*(4, 232) = 0.25, *p* = 0.753, *ε* = 0.45, *η*_*p*_^2^ < 0.01. The interaction found in the latency analysis was further analysed.

When the congruency effect was analysed for each banknote feature separately, the results revealed that the congruency effect was significant in the size-based task, *F*(2, 116) = 47.10, *p* < 0.001, *ε* = 0.82, *η*_*p*_^2^ = 0.45. The RTs in the congruent size condition differed from the RTs in the incongruent size condition, *t*(58) = − 9.70, *p* < 0.001, *d = *0.59, and the neutral size condition, *t*(58) = -5.09, *p* < 0.001, *d* = 0.31. The difference between the neutral size condition and the incongruent size condition was significant also, *t*(58) = 4.61, *p* < 0.001, *d* = 0.28.

In the colour-based task, the congruency effect was significant, *F*(2, 116) = 6.04, *p* = 0.003, *ε* = 0.99, *η*_*p*_^2^ = 0.09. The RTs in the congruent colour condition differed from the RTs in the incongruent colour condition, *t*(58) = − 3.27, *p* = 0.004, *d* = 0.11, and the neutral colour condition, *t*(58) = − 2.65, *p* = 0.027, *d* = 0.09. The difference between the neutral colour condition and the incongruent colour condition was not significant, *t*(58) = 0.61, *p* = 1.00, *d* = 0.02.

Finally, in the design-based task, the congruency effect was significant, *F*(2, 116) = 32.41, *p* < 0.001, *ε* = 0.89, *η*_*p*_^2^ = 0.36. The difference between the congruent design condition and the incongruent design condition was not significant, *t*(58) = − 2.26, *p* = 0.077, *d* = 0.11*.* Moreover, the RTs in the congruent design condition differed from the RTs in the neutral design condition, *t*(58) = 5.56, *p* < 0.001, *d* = 0.28. The difference between the neutral design condition and the incongruent design condition was significant also, *t*(58) = 7.82, *p* < 0.001, *d* = 0.39.

Additionally, we evaluated possible differences between the three comparison tasks at each level of the congruency factor separately. In the congruent condition, the banknote feature effect was significant, *F*(2, 116) = 15.45, *p* < 0.001, *ε* = 0.81, *η*_*p*_^2^ = 0.21. The performance in the size-based task differed from the colour-based task, *t*(58) = 2.62, *p* = 0.030, *d* = 0.35, and the design-based task, *t*(58) = 5.56, *p* < 0.001, *d* = 0.75. The difference between the colour-based task and the design-based task was significant also, *t*(58) = 2.94, *p* = 0.012, *d* = 0.40.

In the incongruent condition, the banknote feature effect was significant, *F*(2, 116) = 29.03, *p* < 0.001, *ε* = 0.92, *η*_*p*_^2^ = 0.33. The size-based task differed from the colour-based task, *t*(58) = 4.48, *p* < 0.001, *d* = 0.60, and the design-based task, *t*(58) = 7.58, *p* < 0.001, *d* = 0.99. The difference between the colour-based task and the design-based task was significant also, *t*(58) = 3.10, *p* = 0.007, *d* = 0.42.

Finally, in the neutral condition, the banknote feature effect was significant, *F*(2, 116) = 38.33, *p* < 0.001, *ε* = 0.75, *η*_*p*_^2^ = 0.40. The size-based task differed from the colour-based task, *t*(58) = 3.32, *p* = 0.004, *d* = 0.46, and the design-based task, *t*(58) = 8.68, *p* < 0.001, *d* = 0.99. The difference between the colour-based task and the design-based task was significant also, *t*(58) = 5.35, *p* < 0.001, *d* = 0.74.

We conducted additional analyses to evaluate whether the magnitude of facilitation and interference effects differed among the three banknote features. *T* test analyses did not reveal a difference between the magnitude of the facilitation effect (neutral condition minus congruent condition) in the size-based task (47 ms) than in the colour-based task (24 ms), *t*(58) = − 1.71, *p* = 0.267, *d* = 0.33. However, the magnitude of the facilitation effect was greater in the size-based task than in the design-based task (− 47 ms), *t*(58) = − 7.13, *p* < 0.001, *d* = 0.99. Finally, the magnitude of the facilitation effect was greater in the colour-based task than in the design-based task, *t*(58) = − 5.42, *p* < 0.001, *d* = 0.99. Regarding the interference effect (neutral condition minus incongruent condition), the magnitude of the interference effect was greater in the size-based task (− 42 ms) than in the colour-based task (− 6 ms), *t*(58) = − 2.94, *p* = 0.012, *d* = 0.54. Furthermore, the magnitude of the interference effect was greater in the design-based task (− 67 ms) compared to the colour-based task, *t*(58) = − 4.89, *p* < 0.001, *d* = 0.90, but showed no difference with the size-based task, *t*(58) = 1.95, *p* = 0.160, *d* = 0.36.

## Discussion

The banknotes are pieces of paper that are legal tender in a country or region and are intended to represent different economic values. Banknotes, along with coins, are cash that people use in their daily lives to engage in economic transactions. The banknotes vary between currencies (euros, dollars, British pound, Chinese renminbi, etc.). Within the same circulating currency, banknotes that represent different economic amounts usually differ from each other mainly in three physical features: size, color and design. Earlier studies have examined the role of physical size when people estimate the monetary value of banknotes (e.g., Ruiz et al., 2017). However, to our knowledge, there is no previous research evaluating together the relative weight of size, color and design of banknotes in monetary tasks (e.g., comparison of the economic value of banknotes). In our study, we addressed this issue directly. We examined the possible facilitation effect derived from having the correct value of a physical feature (i.e., congruent condition) and the possible interference effect of processing banknotes with an incorrect value in that feature (i.e., incongruent condition) compared to a situation where the physical feature under study was not informative (i.e., neutral condition). The analysis of these effects would allow to determine the relevance of size, color and design when people compared the monetary value of banknotes.

The results obtained in our study seem to indicate that the order of relevance of the banknotes' physical features to know their economic value are the size, followed by the color and finally the design. This conclusion stems from two observations. First, the magnitude of the facilitation effect (congruent vs. neutral condition) was higher in the comparison tasks based on size and color than in the one based on the design. While in the neutral condition, the feature under study does not allow the discrimination between banknotes, the congruent condition shows the influence of this feature in the banknote comparison task. Thus, the facilitation effect would be an index of the degree to which the information provided by size, color and design benefits the retrieval of the banknotes' monetary value. On the other hand, in the neutral condition, participants revealed poorer performance in the size > color > design condition. This pattern of outcomes again suggests that size was the most relevant feature for performing the monetary task. Thus, in the neutral size condition, the informational value of this feature was cancelled out, so the participants had to retrieve necessarily the monetary value of the banknotes by analyzing the remaining features (color and design). Consequently, the performance in the neutral size condition would indicate the difficulty of accessing the monetary value of the banknotes due to the impossibility of attending to this feature.

It was striking to observe in our study the same pattern of results in the congruent condition as in the neutral condition across the physical features of the banknotes (i.e., longer response latency in the size > color > design). These differences between features in the congruent condition were not expected since the stimuli in this condition were the same across all three monetary comparison tasks (i.e., pairs of banknotes that maintained the size, color, and design ratios of the actual banknotes). This pattern of results cannot be explained by differences among the participants since they all performed the three comparison tasks. In addition, the order in which the tasks were completed did not affect the participants’ performance. Therefore, the similarity between the congruent, incongruent and neutral conditions seems to suggest that the manipulation of the value of a physical feature (i.e., neutral and incongruent conditions) impacts the way in which the banknotes are processed where they are presented as they are in real life. (i.e., congruent condition). In other words, in a monetary comparison task (e.g., design-based task), the participants would preferentially process the features that are informative in all conditions, congruent, incongruent and neutral (e.g., color and size) at the expense of the less informative feature in that task (e.g., design), because it is predictive of the monetary value of banknotes in only one condition (e.g., congruent condition).

An unexpected finding in our study was obtained in the comparison task based on the design feature. Specifically, the participants did not show facilitation but interference effect with longer response times in the congruent condition than in the neutral condition (47 ms difference). While in the neutral design condition, the design of banknotes was not informative of their monetary value, in the congruent design condition, participants could attend to this feature when performing the task. In addition, regarding the incongruent condition, no differences were observed between this and the congruent condition, but a shorter response time was also observed in the neutral condition compared to the incongruent one. Thus, including a new feature in the congruent condition (i.e., design) hindered the monetary comparison, even though that feature reflected the real design of the banknotes. This pattern of results seems to indicate that the design is the most difficult feature to process compared to the size and color of banknotes. This observation would be supported by the fact that, in general, people have a greater facility for global vs. local perception of visual stimuli (global precedence, Navon, 1977). This global precedence would entail a more efficient processing of size and color compared to the design of the banknotes. In particular, size and color discrimination can be carried out by a holistic inspection of the banknotes, while a monetary comparison based on the banknote design would involve the careful analysis of the image details (differences between the architectural style of gates and windows printed on euro banknotes).

Thus, the outcomes of our study suggest that the analysis of the design feature seems to make difficult the retrieval of the economic value of the banknotes. On the contrary, size and color would be relevant features to perform monetary comparison tasks. However, at this point, we could question why the size of the banknotes turned out to be the most relevant feature. The answer to this issue seems to lie in the fact that both the monetary value and the size of the banknotes refer to the same type of semantic content (i.e., magnitude information). In fact, the facilitation and interference effects observed in the size-based comparison task resemble those found in numerical tasks where the physical size of numbers is evaluated (e.g., number-size congruency effect, Besner & Coltheart, 1979; Henik & Tzelgov, 1982; Santens & Verguts, 2011). In these studies, the participants' performance is less efficient when the size and the numerical magnitude of number pairs are incongruent (e.g., 8–2) compared to congruent number pairs in which the size and the numerical magnitude point in the same direction (e.g., 8–2). The origin of this type of congruency effect has been widely discussed, but the existence of an interaction between the different continuous magnitudes has to be assumed somewhere along their processing stream (Reike & Schwarz, 2017; Santens & Verguts, 2011). Therefore, this relationship could explain the results observed in the number-size congruency effect and also can help us to understand why the congruency effect between monetary value and the size was larger than the two other physical features explored in this study (Leibovich et al., 2017; Lourenco et al., 2016).

The current study has practical implications for the issuance of legal tender (i.e., banknotes in circulation). The results of our work suggest that banknotes denoting different economic amounts should ideally differ both in size and color since both physical features facilitate monetary comparisons when they contain relevant information (i.e., congruent condition) versus when they do not (i.e., neutral and incongruent condition). In addition, the most significant feature that should be present in circulating currency would be size since it facilitates the performance of monetary task to a greater extent than the color feature. On the other hand, the processing of banknote designs produces interference rather than facilitation when people compare banknotes that keep the design of banknotes in real life (congruent condition) relative to the comparison of banknotes without any design (neutral condition). It should be taken into account that this research has been carried out with a specific currency, the Euro, whose banknotes have specific physical features. Therefore, it would be interesting to be able to extend this research in order to observe not only how the familiarity with the currency would influence the pattern of results observed, but also what would happen with those currencies whose banknotes have no difference in some of the physical features examined. Therefore, from the study reported here, it would be advisable to put into circulation banknotes with the same design but variability in size and color depending on the amount of money banknotes represent.

## Data Availability

To comply with APA Ethics Code Standard 8.14a (sharing research data for verification), the stimuli, experimental procedure, data and analyses reported in this manuscript have been stored in a freely accessible repository (reference links have been indicated in the manuscript).
